# The Actin Cytoskeleton: A Mechanical Intermediate for Signal Integration at the Immunological Synapse

**DOI:** 10.3389/fcell.2018.00116

**Published:** 2018-09-19

**Authors:** Nathan H. Roy, Janis K. Burkhardt

**Affiliations:** ^1^Department of Pathology and Laboratory Medicine, Children's Hospital of Philadelphia Research Institute, Philadelphia, PA, United States; ^2^Perelman School of Medicine at the University of Pennsylvania, Philadelphia, PA, United States

**Keywords:** immunological synapse, integrin, TCR, actin, force, mechanotransduction

## Abstract

The immunological synapse (IS) is a specialized structure that serves as a platform for cell-cell communication between a T cell and an antigen-presenting cell (APC). Engagement of the T cell receptor (TCR) with cognate peptide-MHC complexes on the APC activates the T cell and instructs its differentiation. Proper T cell activation also requires engagement of additional receptor-ligand pairs, which promote sustained adhesion and deliver costimulatory signals. These events are orchestrated by T cell actin dynamics, which organize IS components and facilitate their signaling. The actin network flows from the edge of the cell inward, driving the centralization of TCR microclusters and providing the force to activate the integrin LFA-1. We recently showed that engagement of LFA-1 slows actin flow, and that this affects TCR signaling. This study highlights the physical nature of the IS, and contributes to a growing appreciation in the field that mechanosensing and mechanotransduction are essential for IS function. Additionally, it is becoming clear that there are multiple types of actin structures at the IS that promote signaling in distinct ways. How the different actin structures contribute to force production and mechanotransduction is just beginning to be explored. In this Perspective, we will feature recent work from our lab and others, that collectively points toward a model in which actin dynamics drive mechanical signaling and receptor crosstalk during T cell activation.

## Introduction

Activation of T cells by antigen presenting cells (APCs) takes place at a specialized cell-cell interface termed the immunological synapse (IS). Initial IS formation is driven by interaction of the T cell receptor (TCR) and accessory molecules on the T cell surface with peptide-MHC complexes and other cognate ligands on the APC. These interactions trigger large cytoskeletal changes at the IS along with the reorganization of many molecules, including the TCR, adhesion receptors, kinases, phosphatases, adapters, and other signaling intermediates (Dustin et al., 2010). Signaling events that take place at the IS lead to initial activation of naïve T cells and direct their subsequent lineage commitment. For effector cells, signaling at this interface induces polarized release of stimulatory cytokines or cytotoxic granules. The APC side of the IS is also a site of active signaling, directing differentiation events that promote immune responses or tolerance. Thus, the IS acts as a highly tunable signaling platform that integrates spatial, mechanical, and biochemical signals. How the different signals are interpreted to produce the desired cellular responses is an area of intense research, with implications for the development of therapeutics targeting cancer and autoimmunity.

**Figure 1:**
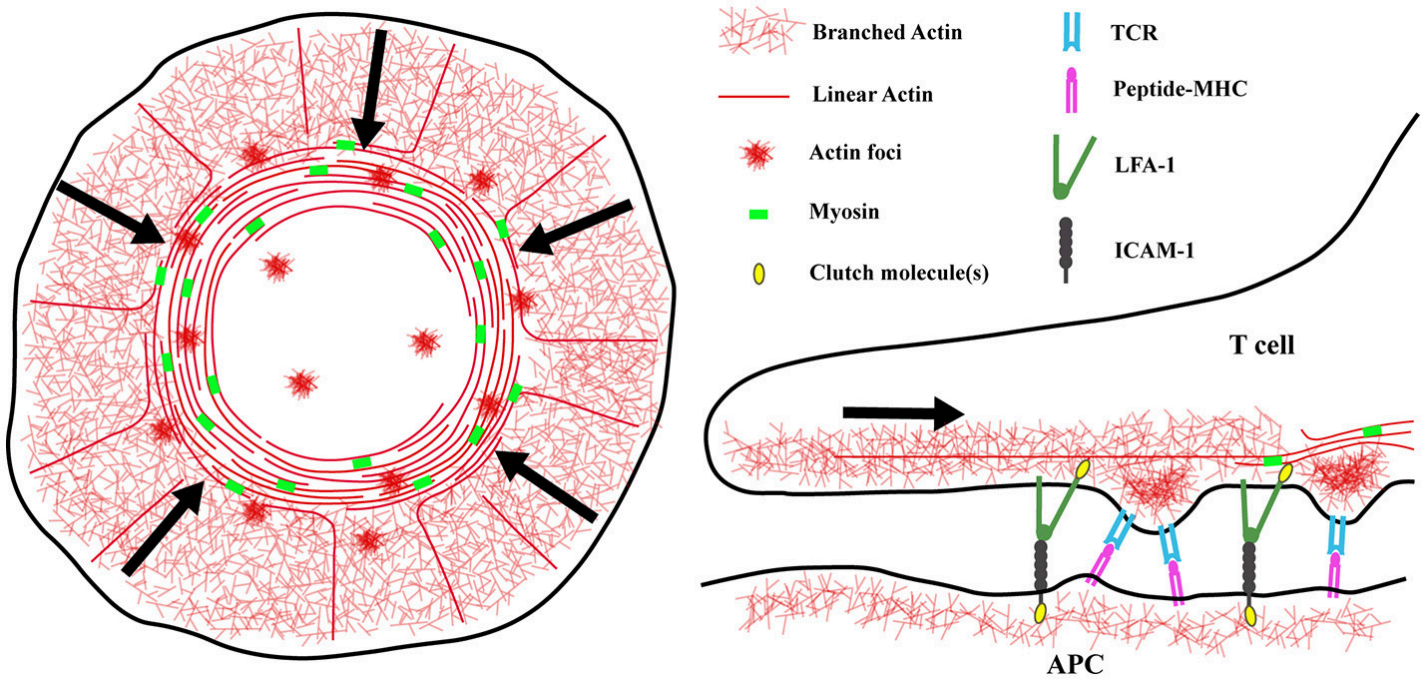
Actin architecture at the immunological synapse. **(Left)** Diagram showing the distinct actin networks at the immunological synapse. The architecture of a spreading T cell can be divided into three zones, a peripheral region enriched in branched actin filaments corresponding to the lamellipodium of a migrating cell, an inner lamellar region containing acto-myosin arcs, and a central actin-poor region. At the synapse edge, WAVE2 and Arp2/3 complex direct the polymerization of branched actin filaments, pushing the network to flow inward. Also in this region, formins nucleate linear actin filaments that are then organized into concentric arcs by myosin activity. Lastly, WASp dependent actin “foci” appear at the synapse and co-localize with TCR microclusters. **(Right)** The different networks may have different functions at the IS. Actin foci are thought to protrude from the T cell into the APC membrane and facilitate TCR/peptide-MHC interactions. The rapidly flowing branched actin network, and to a lesser extent the slower moving actomyosin arcs, provide the force needed to separate the cytoplasmic tails of LFA-1 and transition it into the high affinity state. Actin arcs are also important for sweeping TCR microclusters inward and promoting T cell activation. Understanding the details of how these different networks transmit mechanical energy will have important implications for mechanosensing and mechanotransduction at the IS.

The actin cytoskeleton plays a crucial role in the formation and maintenance of IS structure. Although there are numerous variations, the cannonical IS resembles a “bullseye,” consisting of concentric rings with distinct protein and lipid compositions. A branched actin network is found within the outermost region, which corresponds to the lamellipodium of a migratory cell. Moving inward, the integrin LFA-1 on the T cell and its ligand ICAM-1 on the APC form an inner (lamellar) ring that surrounds a central, actin poor region (Dustin et al., 2010). Upon antigen engagement, TCRs and other early signaling molecules form nanometer-scale microclusters where early signaling events take place. These microclusters initially form near the edge of the IS and move inward, finally accumulating in the central region where signals are extinguished (Varma et al., 2006). Actin dynamics are important for multiple aspects of IS function, including initial cell spreading, TCR microcluster mobility, downstream signaling, and cytotoxic granule release (Billadeau et al., 2007; Kumari et al., 2014). Live cell microscopy has revealed that the actin network “flows” from the outer edge of the synapse inward, sweeping in TCR microclusters and providing force to activate LFA-1 (Bunnell et al., 2001; Babich et al., 2012; Yi et al., 2012; Comrie et al., 2015a) (Figure 1). This actin flow is driven mostly by actin polymerization, although there is a contribution from myosin motors (Babich et al., 2012; Yi et al., 2012). Importantly, halting actin flow with pharmacological inhibitors immediately inhibits downstream signaling. This finding demonstrates that F-actin does not function simply as a scaffold for assembly of signaling complexes. Instead, continued actin flow is required for proper IS function (Babich et al., 2012). It seems clear that the requirement for continued actin flow represents the need for actin-dependent force on early signaling intermediates associated with T cell activation. In addition to LFA-1, which is known to undergo force-dependent conformation change, there is strong evidence that the TCR itself is a mechanosensor (Kim et al., 2009; Liu et al., 2014; Das et al., 2015), as are several intracellular signaling molecules such as talin and the p130Cas homologue CasL (Janoštiak et al., 2014; Yan et al., 2015). Recently, it has become clear that mechanical and biochemical signals at the IS are integrated by actin dynamics. This Perspective highlights recent work from our lab and others that has shed new light on this biology.

### Coupling of integrins to the actin network creates a tug-of-war at the IS

**Figure 2:**
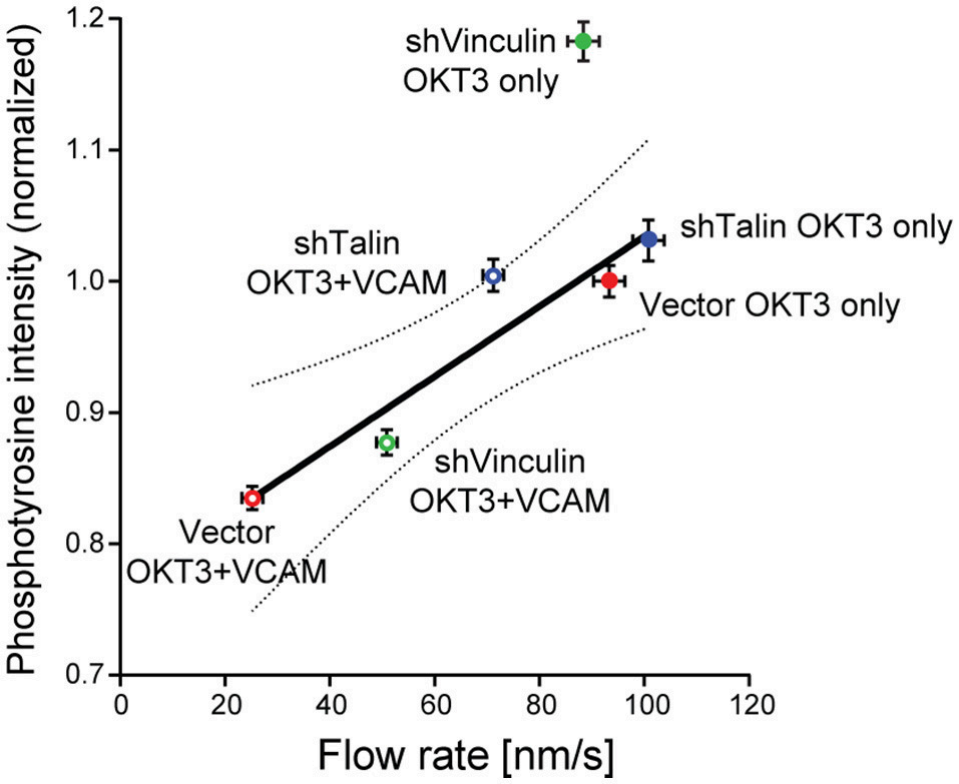
Correlation between actin flow rates and tyrosine phosphorylation during T cell activation. Results from two types of experiments were combined to assess how actin flow rates affect TCR signaling. Jurkat T cells stably expressing GFP-actin were transduced with recombinant lentiviruses expressing shRNAs specific for vinculin or talin, or with an empty control vector. Cells were selected with puromycin and suppression was verified by immunoblotting. Cells were then allowed to spead on glass coverslips coated with 10 μg/ml anti-TCR (OKT3, BioXCell), alone or in combination with 2 μg/ml VCAM-1 Fc (R&D Systems), and imaged by spinning disk confocal microscopy. Actin flow rates within the lamellipodial region were determined by kymographic analysis as detailed in (Jankowska and Burkhardt, 2017). In parallel studies, cells were allowed to spread on stimulatory coverslips for 5 min, fixed, permeabilized, and labeled for phosphotyrosine (PY20, directly conjugated to Alexafluor-488 using the Mix-n-Stain kit from Biotium). Cells were imaged by confocal microscopy (50–60 randomly selected cells per condition, z-stacks of 3-planes spaced 0.25 μm apart), and the integrated signal intensity from the rendered stacks was measured using Volocity 6.3 by manually drawing a ROI around each cell. Average phosphotyrosine signal per cell was then plotted as a function of actin flow rates. In vector-transduced control cells, VCAM-1 engagement slows the actin flow rate and decreases phosphotyrosine signaling. Knocking down the clutch molecules talin or vinculin relieved the slowing effect of integrin engagement, suggesting that mechanical drag is at least partly responsible for the slowing of the actin network. Regression analysis revealed a linear correlation between actin flow rates and phosphotyrosine signaling, with *R*^2^ = 0.90. Dotted line shows the 95% confidence interval. Data represent means ± SEM from three independent experiments. Note that shVinculin on OKT3 falls outside this confidence interval, suggesting an additional function of vinculin beyond a clutch in this system. These data show that the actin network serves as a mechanical intermediate through which integrins modulate TCR signaling. Adapted from Jankowska et al. (2018).

By acting as direct links between the cytoskeleton and the extracellular environment, integrins are well-positioned to act as points of traction for force transduction as well as to relay mechanical information about the environment to the cell interior. In non-hematopoietic cells, integrins are known to bind indirectly to the actin cytoskeleton, via so called “clutch proteins” (Case and Waterman, 2015). This regulated linkage allows the cell to transfer some of the mechanical energy generated by actin dynamics to the underlying substrate. As a result of this mechanical coupling, this process slows retrograde actin flow (Hu et al., 2007; Owen et al., 2017). At the IS, the integrins LFA-1 (αLβ2), and VLA-4 (α4β1) on the T cell surface interact with ICAM-1 and VCAM-1 on the APC, respectively, and these interactions help to stabilize the IS interface. Since T cells exert very low traction forces (in the pN range Bashour et al., 2014; Hui et al., 2015; Liu et al., 2016) and lack obvious focal adhesions, it was unclear to what extent LFA-1 engagement would influence actin flow at the IS, and whether this would impact downstream signaling. One early study suggested that VLA-4 engagement slowed actin flow in a T cell line (Nguyen et al., 2008), but the question had not been explored further. Directly addressing this question, a recent study using primary T cells found that engagement of either LFA-1 or VLA-4 significantly slows TCR-induced actin flow (Jankowska et al., 2018). Importantly, this slowing was distributed across the actin network, microns away from the adhesion sites, and was dependent upon the clutch molecules talin and vinculin (Jankowska et al., 2018). These data strongly suggest that drag created by clutched integrins can globally affect actin retrograde flow rates. Thus, one can think of the actin network at the IS as a single mechanical unit, where perturbations that affect the mechanical properties of one area of the network can propagate to surrounding areas and influence distant force-sensitive proteins. In keeping with this idea, with only one exception^1^, we found a linear correlation between the magnitude of tyrosine phosphorylation at the IS, and actin flow rates under a variety of experimental conditions (Figure 2). Interestingly, Ca^2+^ responses were unaffected by integrin engagement, supporting a 3-step model: (1) TCR engagement induces initial signaling events, including Ca^2+^ influx, leading to actin polymerization at the IS (Hartzell et al., 2016) (2) Actin polymerization and resulting centripetal flow drives force-dependent signal amplification, resulting in robust and sustained tyrosine phosphorylation. (3) Engaged integrins resist these forces, slowing actin flow, and diminishing the magnitude of TCR-dependent tyrosine phosphorylation.

But how, exactly, does actin promote signal amplification? It is clear that actin flow can drive the centralization of microclusters and help organize other components of the IS, but the mechanisms underlying mechanosensing and mechanotransduction at the IS are just being elucidated, and the details are controversial. As previously mentioned, the TCR itself is thought to be a mechanosensitive complex (Kim et al., 2009; Liu et al., 2014; Das et al., 2015), and TCR signaling depends on force produced by the actin network (Hu and Butte, 2016). Additionally, force production through the actin network seems to aid in peptide discrimination, a central part of adaptive immunity (Das et al., 2015; Liu et al., 2016). The ability to discriminate between peptides with different affinities or binding kinetics likely arises from the catch bond behavior of TCR—peptide-MHC interactions (Liu et al., 2014; Das et al., 2015). Catch bonds display increased bond lifetime with applied force (until a maximum is reached before bond severing), ensuring that only high affinity interactions will have the long lifetimes needed to initiate appropriate downstream signaling. Catch and slip bonds (bonds where binding decreases with force) are better studied for integrins, where they play an important role in mechanosensing. For integrins, it is clear that the force loading rate, and not simply the application of force *per se*, is a fundamental factor (Elosegui-Artola et al., 2018). At the IS, different actin flow rates would equate to different loading rates, and could influence the function of mechanosensitive proteins such as the TCR and integrins. Importantly, loading rate is not only dependent upon the applied force, but also the substrate rigidity. For a T cell, the relevant substrate is the surface of an interacting APC. Therefore, the mechanical properties of both the T cell and APC contribute to T cell mechanosensing. This interplay highlights the complexity of cell-cell signaling at the IS, and represents another layer of regulation employed by T cells and APCs to tune activation. Interestingly, dendritic cells alter their rigidity after maturation [(Bufi et al., 2015) and our unpublished data], and T cells display differential responses when activated on substrates of varying stiffness (Judokusumo et al., 2012; O'Connor et al., 2012; Lambert et al., 2017; Saitakis et al., 2017), indicating that this mechanical crosstalk is biologically relevant.

Because of its strong coupling to the actin cytoskeleton, LFA-1 has provided a unique opportunity to monitor mechanosensing at the IS. LFA-1 transitions through three distinct conformations, spanning a 1000-fold range in binding affinity for ICAM-1 (Springer and Dustin, 2012). Generation of the high affinity extended open LFA-1 conformation requires separation of the α and β chain cytoplasmic tails, a process that depends on the actin cytoskeleton (Zhu et al., 2008; Schürpf and Springer, 2011; Li and Springer, 2017). A recent study utilizing a panel of antibodies specific for the different LFA-1 conformations revealed that actin flow is essential to produce the force necessary to transition LFA-1 into the extended open conformation at the IS (Comrie et al., 2015a). Interestingly, linkage of ICAM-1 to the APC cytoskeleton was also required to produce optimal counter-force on LFA-1. ICAM-1 mutants that were mobile in the membrane showed reduced ability to induce high affinity LFA-1, resulting in diminished T cell adhesion and activation (Comrie et al., 2015b). These data support a model in which binding of LFA-1 to ICAM-1 generates a direct mechanical link between the cytoskeleton of the T cell and the cytoskeleton of the APC. Functionally, this means that the actin cytoskeleton in the APC could influence mechanosensing and mechanotransduction in the T cell, and vice versa. Additionally, this sets up an interesting case at the IS where changes in ICAM-1 mobility on the APC can in turn create drag on clutched LFA-1 in the T cell, thus slowing actin flow and modulating TCR signaling. This is another instance in which actin flow on the T cell side of the IS works in concert with the biophysical properties of the APC to determine the force applied on mechanosensitive proteins. Thus, ligand mobility, like APC stiffness, can modulate T cell mechanotransduction. And just as DCs become stiffer upon maturation, they also show reduced mobility of T cell ligands such as ICAM-1 (Comrie et al., 2015b). Maturation-associated changes in the DC actin cytoskeleton undoubtedly play a key role in determining these biophysical properties, and understanding how these properties are regulated by the APC will yield new information about mechanical signaling at the IS.

### Forces within the T cell are driven by multiple actin networks

While most of the research on actin responses at the IS have focused on the prominent lamellipodial ring of branched actin filaments, it has recently become clear that there are actually several actin networks at the IS, organized by distinct nucleation promoting factors (Figure 1). The two main classes of nucleation promoting factors at the IS are members of the WASp/SCAR family (WAVE2 and WASp), which activate the Arp2/3 complex to generate branched actin filaments, and formins, which generate linear actin filaments. WAVE2 localizes to the peripheral actin ring, where it is responsible for the formation of the lamellipodial branched actin network (Nolz et al., 2006; Le Floc'h et al., 2013). Formins nucleate long linear actin filaments at the cell periphery (Murugesan et al., 2016). As these filaments move inward, myosin activity organizes them into concentric actin arcs, aiding in the centralization and signaling of TCR microclusters. Lastly, WASp generates small actin patches, dubbed “foci,” at sites of TCR engagement (Kumari et al., 2015). Importantly, nucleation promoting factors constantly compete with each other for free actin monomers and other factors (Burke et al., 2014; Rotty and Bear, 2014; Lomakin et al., 2015; Suarez et al., 2015; Davidson et al., 2018). Thus, inhibition of the Arp2/3 complex augments production of formin-driven structures (Murugesan et al., 2016). Complicating matters further, actin polymerization by different NFPs is triggered by different molecules at the IS. Ligation of the TCR drives Arp2/3 based actin polymerization, while the LFA-1/ICAM-1 interaction results in formin dependent polymerization (Tabdanov et al., 2015). Moreover, there is evidence that the different actin networks generated by IS-associated nucleation promoting factors have specific functions. WAVE2, but not WASp, is essential for T cell spreading, and WAVE2 also plays an important role in regulating integrin-dependent adhesion (Cannon and Burkhardt, 2004; Nolz et al., 2006, 2007). In contrast, WASp-induced actin foci likely correspond to TCR-mediated protrusions that function to overcome glycocalyx barriers and promote TCR-pMHC contacts (Kumari et al., 2015; Cai et al., 2017). These structures are dispensable for activation of the most TCR-proximal phosphorylation events, but are required for efficient PLCγ1 activation. Thus, WASp deficient T cells, which lack actin foci but have a normal peripheral actin ring, exhibit defects in calcium release downstream of TCR activation (Kumari et al., 2015). Finally, myosin activity appears to be important for maintaining IS symmetry. While myosin function is dispensable for T cells responding to strong stimuli (Babich et al., 2012), inhibiting myosin activity or formin function perturbs the formation of actomyosin arcs, and affects the ability of T cells to respond to low affinity ligands (Hong et al., 2017).

While general rules are emerging about how the different dynamic actin networks contribute to T cell activation, it is not yet clear exactly how they promote mechanosensing and mechanotransduction at the IS. A recent study by Liu et al. using fluorescent tension sensors shows that actin polymerization, but not myosin contractility, is required for force production through the TCR (Liu et al., 2016). Surprisingly, inhibition of the Arp2/3 complex did not result in a decrease in force production, but instead an increase was observed. The authors also inhibited the upstream Rho GTPases Rac and Cdc42 and found that only Cdc42 activity was required for force production (Liu et al., 2016). These findings are in line with the idea that the different actin networks differentially contribute to force production and mechanosensing. Importantly, these distinct actin networks, which are formed in response to distinct receptor stimuli, are mechanically interconnected. For example, costimulation through CD28 enhances forces exerted by the TCR (Bashour et al., 2014). Similarly, slowing of actin flow by engagement of the β1-integrin VLA-4 diminishes force-dependent conformational change of the β2-integrin LFA-1 (Comrie et al., 2015a). While the field has so far focused on TCR and integrins, there is also evidence that the different actin networks differentially influence other mechanosensitive proteins in the TCR signaling pathway. For instance, the Cas family proteins are documented mechanosensitive proteins (Janoštiak et al., 2014). Upon force application, they undergo an accordion-like structural change, exposing multiple phosphorylation sites (Sawada et al., 2006). CasL is expressed in hematopoietic cells and is localized to the IS in its activated, phosphorylated state. Inhibiting either the Arp2/3 complex or myosin activity results in decreased phosphorylation of CasL (Kumari et al., 2012; Yu et al., 2012; Santos et al., 2016; Hong et al., 2017), opposite to what has been seen for the TCR. Finally, these concepts extend to additional force-dependent processes at the IS. For example, in cytotoxic T cells, forces exerted by the T cell create membrane tension on the target cell, resulting in optimal target cell killing. Although the specific nucleation promoting factors for this force production are unknown, the process involves PI3K signaling, and the Rac GEF Dock2 plays a role (Basu et al., 2016). A main goal moving forward will be to tease apart how the different actin regulatory pathways contribute to discrete mechanical processes at the IS.

## Conclusions and future outlook

The immunological synapse is a highly mechanical signaling platform. Because mechanical signaling relies on the biophysical properties of both the T cell and the APC, future work aimed at understanding the mechanical crosstalk between these entities will help provide a more complete picture of signaling at the IS. In addition to determining the relevant cytoskeletal factors required for mechanotransduction, identification of novel mechanosensors is also a priority. While the field has tended to focus on cell surface receptors, it is likely that many IS-associated proteins can change structure in response force and thus act as mechanosensors. Recently, the activity of the phosphatase SHP-1 was shown to be regulated via a force-induced structural change at the NK cell cytotoxic synapse (Matalon et al., 2018). The force necessary to produce this change results from actin polymerization, but also the ability of SHP-1 to bind to dynamic actin filaments. As the field progresses and additional mechanosensors are identified, it will be important determine which actin networks regulate their activity, and how these mechanical signals are integrated. Lastly, some of these same questions apply to other aspects of T cell biology, such as extravasation. During extravasation the T cell and endothelial cell form an intimate, dynamic cell-cell contact, ultimately leading to diapedesis through the endothelial layer. Analysis of this process has revealed that the T cell sends actin driven podosome-like protrusions into the endothelial cell surface, and it has been hypothesized that these structures serve as tactile fingers to mechanically probe the underlying endothelia (Carman et al., 2007; Martinelli et al., 2014). These protrusions resemble invadopodial structures that have been described in other systems, most notably in cancer cells, where they have been shown to mediate mechanosensing and tissue invasion (Eddy et al., 2017). Thus, understanding which actin structures drive force production and identifying the relevant mechanosensors will provide valuable information into how cells sense environmental cues and launch appropriate responses.

## Author contributions

All authors listed have made a substantial, direct and intellectual contribution to the work, and approved it for publication.

## Conflict of interest statement

The authors declare that the research was conducted in the absence of any commercial or financial relationships that could be construed as a potential conflict of interest.

## Footnotes

^1^The one exception to this correlation is in T cells suppressed for vinculin and responding to anti-TCR alone. These cells consistently exhibit increased tyrosine phosphorylation without increased actin flow. This suggests that in addition to its role as a clutch protein, vinculin dampens TCR signaling via a mechanism that does not involve integrin-dependent effects on actin dynamics. The basis for this latter mechanism is unclear, but as we observed similar effects of vinculin suppression on ZAP-70 phosphorylation (Jankowska et al., 2018), the mechanism is likely TCR proximal.
